# Short Chain Fatty Acids Commonly Produced by Gut Microbiota Influence *Salmonella* *enterica* Motility, Biofilm Formation, and Gene Expression

**DOI:** 10.3390/antibiotics8040265

**Published:** 2019-12-13

**Authors:** Alexandre Lamas, Patricia Regal, Beatriz Vázquez, Alberto Cepeda, Carlos Manuel Franco

**Affiliations:** Laboratorio de Higiene Inspección y Control de Alimentos, Departamento de Química Analítica, Nutrición y Bromatología, Universidad de Santiago de Compostela, 27002 Lugo, Spain; patricia.regal@usc.es (P.R.); beatriz.vazquez@usc.es (B.V.); alberto.cepeda@usc.es (A.C.); carlos.franco@usc.es (C.M.F.)

**Keywords:** *Salmonella*, short chain fatty acids, antimicrobial activity, biofilm, motility, gene expression

## Abstract

Short chain fatty acids (SCFAs) are commonly produced by healthy gut microbiota and they have a protective role against enteric pathogens. SCFAs also have direct antimicrobial activity against bacterial pathogens by diffusion across the bacterial membrane and reduction of intracellular pH. Due to this antimicrobial activity, SCFAs have promising applications in human health and food safety. In this study, the minimum inhibitory concentrations (MICs) of four SCFAs (acetic acid, butyric acid, propionic acid, and valeric acid) in *Salmonella* strains isolated from poultry were determined. The effect of subinhibitory concentrations of SCFAs in *Salmonella* biofilm formation, motility, and gene expression was also evaluated. Butyric acid, propionic acid, and valeric acid showed a MIC of 3750 µg/mL in all strains tested, while the MIC of acetic acid was between 1875 and 3750 µg/mL. Subinhibitory concentrations of SCFAs significantly (*p* *<* 0.05) reduced the motility of all *Salmonella* strains, especially in the presence of acetic acid. Biofilm formation was also significantly (*p* < 0.05) lower in the presence of SCFAs in some of the *Salmonella* strains. *Salmonella* strain. *Salmonella* Typhimurium T7 showed significant (*p* < 0.05) upregulation of important virulence genes, such as *invA* and *hilA*, especially in the presence of butyric acid. Therefore, SCFAs are promising substances for the inhibition of the growth of foodborne pathogens. However, it is important to avoid the use of subinhibitory concentrations that could increase the virulence of foodborne pathogen *Salmonella*.

## 1. Introduction

Short chain fatty acids (SCFAs) are end metabolites produced by microbial fermentation of undigested carbohydrates and dietary fibers. Butyrate, acetate, and propionate are the main SCFAs, but others, such as lactate and valerate, are also produced by microbiota [1,2,3]. SCFAs have important roles in human gut homeostasis by exerting several effects on the host and its own microbiota. SCFAs are used as a source of energy by intestinal epithelial cells. They also modulate the absorption of electrolytes and increase the production of anti-inflammatory cytokines. SCFAs also reduce the production of molecules that act as pro-inflammatory substances, such as nitrous oxide, interleukins, and tumor necrosis factor. SCFAs also have a protective effect against bacterial pathogens by maintaining the integrity of the epithelial barrier [4,5]. In addition, it has been observed that SCFAs induce the production of antimicrobial peptides by enterocytes [6]. In the same way, it has been observed that macrophages that differentiate in the presence of butyrate show increased antimicrobial activity even in the absence of an increased inflammatory cytokine response [6].

In adequate concentrations, SCFAs also have direct antimicrobial activity against pathogenic bacteria. SCFAs can reduce microbial growth by modifying the intracellular pH and the metabolism. At lower pH, SCFAs are commonly present in nonionized forms that can diffuse across the bacterial membrane into the bacterial cytoplasm. Once in the cytoplasm, SCFAs dissociate, increasing the anion and proton concentrations and lowering the intracellular pH [7,8,9].

The production of adequate and balanced SCFAs by a healthy gut microbiota is an important factor that prevents infection by common foodborne pathogens [10]. It was found that *Bacteriodes* spp. mediates resistance to *Salmonella* colonization by producing propionate [9]. Diverse studies have observed total SFCAs concentrations ranging between 60 and 85 mM with levels of acetate between 40 and 50 mM, propionate around 15 mM, and butyrate around 10 mM [11,12]. A dysregulation of SCFAs levels can facilitate the colonization of intestine by pathogens. In this sense, decreased concentrations of butyrate cause upregulation of virulence genes in enterohemorrhagic *Escherichia coli* (EHEC), and different spatial gradients of SCFAs regulate the expression of virulence and commensal genes in *Campylobacter jejuni* [13,14]. Propionate decreases the expression of *Salmonella* genes located in *Salmonella* Pathogenicity Island 1 (SPI-1) [15]. In addition, pre-incubation of *Salmonella enteritidis* with propionate and butyrate results in a reduction in host cell invasion [16]. It is noteworthy that SCFA concentrations similar to those found in the distal ileum cause upregulation of *Salmonella* virulence genes, while concentrations similar to those found in the colon have the opposite effect [17]. Therefore, the protective and antimicrobial effects of SCFAs are concentration dependent.

Understanding the inhibitory effects of SCFAs on enteric pathogens is not only important from a gut health point-of-view, but this knowledge can also be important from a food safety point-of-view. In the last years, different researchers have been evaluating alternatives to inhibit the growth of foodborne pathogens in the food industry as antimicrobial peptides [18,19]. In the same way, SCFAs can be added to food and feed as preservatives, avoiding the growth of bacterial pathogens. The SCFAs ingested through can also have positive effects in gut balance with inhibitory effects in enteric pathogens. For this purpose, the aim of this study was to determine the minimum inhibitory and biocidal concentrations of four SCFAs (acetic acid, butyric acid, propionic acid, and valeric acid) in *Salmonella* and to determine the effect of subinhibitory concentrations of these SCFAs in biofilm formation, motility, and gene expression. As poultry products are mainly responsible of human salmonellosis [20], the authors decided to use a total of 12 *Salmonella* strains isolated from poultry. These *Salmonella* strains also belonged to seven different serotypes and two *Salmonella* subspecies in order to observe if they have a similar response to the presence of SCFAs.

## 2. Results and Discussion

### 2.1. Antimicrobial Activity of Short Chain Fatty Acids

Until now, there have been limited studies evaluating the MIC and minimum biocidal concentration (MBC) values of SCFAs in food-borne pathogens [21]. In this study, the MICs of the four SCFAs were determined. Butyric acid, propionic acid, and valeric acid had the same MIC value (3750 µg/mL) in all *Salmonella* strains tested in this study. In addition, all strains showed the same MBC (3750 µg/mL) with all the SFCAs tested. However, in the case of acetic acid, five of the twelve strains had a MIC value of 1875 µg/mL, while the other strains had the same MIC as those with the others SCFAs (3750 µg/mL). The results observed with acetic acid were similar to those observed in previous work where the MIC_50_ was 1650 µg/mL and the MIC_90_ was 3280 µg/mL in 88 multidrug resistant *Salmonella* isolates [22]. In the same way, another study that evaluated the antimicrobial activity of acetic acid in different pathogens, such as *E. coli, Staphylococcus aureus,* or *Acinetobacter baumannii,* found that the MIC values were between 0.16% and 0.31% [23]. However, another study that determined the antimicrobial activity of acetic acid in *E. coli* and *Salmonella* sp. strains found MIC values of 1.5% and 1%, respectively [24]. *Campylobacter coli* strains isolated from pigs had MIC values of 2048 µg/mL in most of the strains tested in the presence of butyric acid and propionic acid [25], lower than the MIC values observed in this study. Conversely, in others works with *Salmonella* Typhimurium ATCC 14028 or *Vibrio harveyi*, the inhibition values and propionic acid of butyric acid, respectively, were between 500 and 100 µg/mL [26,27]. The antimicrobial activity of SCFAs is due to their diffusion across the bacterial membrane into the bacterial cytoplasm, modifying the intracellular pH and the metabolism [7,8].

### 2.2. Effect of Short Chain Fatty Acids in Salmonella enterica Motility

Once the MICs were calculated, the effect of SCFAs on *Salmonella* motility was determined. For this purpose, the highest subinhibitory concentrations of the SCFAs were used. The motility was significantly reduced in the presence of SCFAs in comparison to the control semisolid agar in all strains tested in this study (Table 1). There were also significant differences (*p* < 0.05) between the different SCFAs included in the study. The higher reduction in motility was observed in the presence of acetic acid, while valeric acid caused a lower reduction in motility in comparison to the control agar. In accordance with this study, a previous study also observed that organic acids, including acetic acid, decreased the motility of *E. coli* and *Salmonella* strains isolated from fresh fruit and vegetables [24]. It was observed that *Salmonella* Typhimurium ATCC 14,028 motility decreased as the subinhibitory concentrations of propionic acid increased [27]. Deepening in these effects, it has been observed that the fermentation products of *Clostridium ramosum* and SCFAs reduce the motility in enterohemorrhagic *E. coli* (EHEC), disturb the flagellar rotation, and the flagella length is lower in comparison to control conditions [28]. However, the decrease in intracellular pH could be responsible for the slow flagella motor rotation and the reduced motility [29].

### 2.3. Effect of Short Chain Fatty Acids on Biofilm Formation

**Figure 1:**
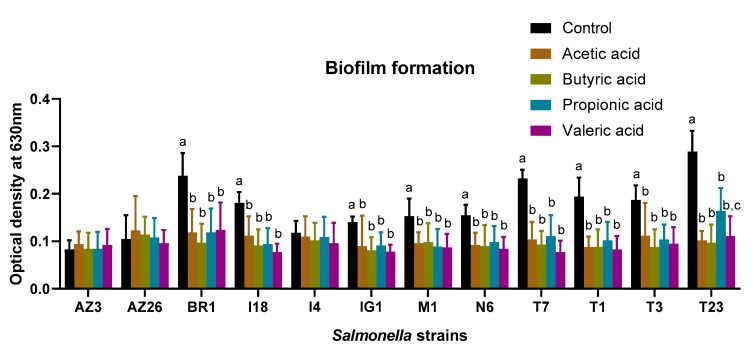
Influence of the four short chain fatty acids tested in this study (acetic acid, propionic acid, butyric acid, and valeric acid) in the biofilm formation of *Salmonella* strains at 37 °C expressed as optical density. Results are expressed as the mean of three different experiments (*n* = 3) and error bars represent the standard deviation. Different letters in the same *Salmonella* strain represent statistically significant differences (*p* < 0.05) between the different growth media.

The presence of subinhibitory concentrations of SCFAs influence biofilm formation in some of the *Salmonella* strains included in this study (Figure 1). There were no significant differences in the reduction of biofilm formation between the different SCFAs tested in eight of the nine strains. However, in *Salmonella* Typhimurium T23, the reduction in biofilm formation caused by propionic acid was significantly lower (*p* < 0.05) than the reduction caused by acetic acid and butyric acid. It is also worth noting that the strains in which biofilm formation was not influenced by the SFCAs were *Salmonella* strains with a low ability to produce a biofilm at 37 °C.

Consistent with the above-mentioned, previous studies have also observed a reduction in biofilm formation by *Salmonella* strains under the presence of subinhibitory concentrations of acetic acid [23,24,27]. The presence of acetic acid causes inhibition of extracellular polysaccharides production in foodborne pathogens such as *E. coli* or *S.* Typhimurium, although to a lesser extent in the latter [24]. Acetic acid also showed anti-quorum sensing activity in *E. coli* and *S.* Typhimurium [24]. Quorum sensing is a cell-to-cell communication mechanism that has great importance in biofilm formation, and inhibition of this mechanism can result in lower biofilm formation [30]. Flagella also have important roles in the initial adhesion of bacterial cells to a surface in biofilm formation [31]. In this sense, it has been observed that propionic acid causes changes in type 1 fimbriae, with a brittle and broken appearance [27]. The authors hypothesize that these effects of SCFAs, in combination with the short flagella that can be synthetized in the presence of intracellular acid [29], could be responsible for the lower biofilm formation observed. The results of this study should be considered as preliminary and only exploratory in nature and might not have external validity. There are some biofilm formation assays that are more labor-intensive and more accurate than using 96-well microplates in recovering the sessile cells using sonication or the beads/vortex method [32,33]. Therefore, the preliminary results of this study should be confirmed in the future by using more accurate assays and including different surfaces of great importance in the food industry, such as stainless steel.

### 2.4. Effect of Short Chain Fatty Acids on Salmonella enterica Gene Expression

The results of this and previous studies have demonstrated that SCFAs are able to inhibit the growth of foodborne pathogens such as *Salmonella* [9,22,24,27]. However, previous studies also observed that subinhibitory concentrations of SCFAs can induce the expression of virulence genes in *E. coli* [14], *Salmonella* [17], and *C. jejuni* [13]. In this study, the expression of 14 genes related to virulence, stress response, and carbon storage was determined in two *Salmonella* strains (*S.* Typhimurium T7 and *S.* Infantis I4).

**Figure 2:**
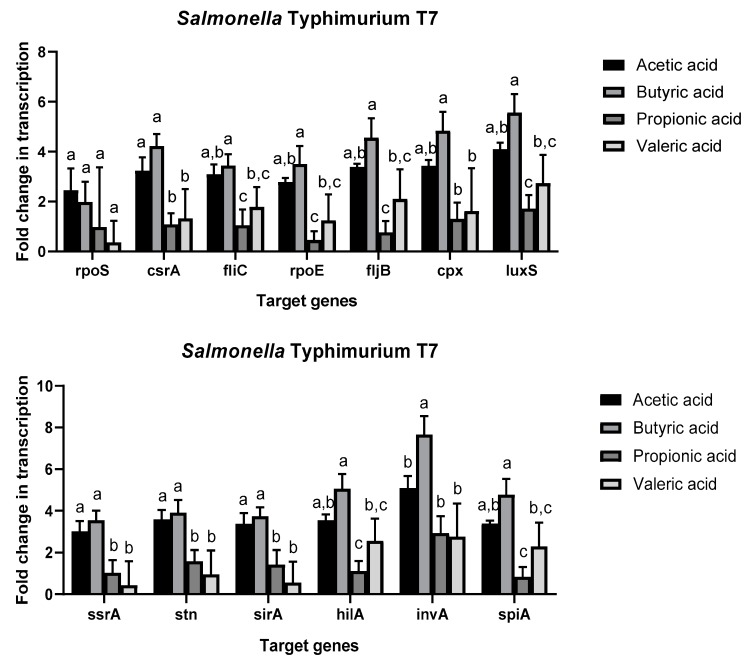
Fold change normalized to control gene *16s rRNA* in the transcription of genes related to virulence and stress in the presence of short chain fatty acids in comparison to control TSB growth media in *Salmonella* Typhimurium T7. Only target genes with different letters between the different growth media present statistically significant differences after analysis of variance (one-way ANOVA) and the Tukey’s honestly significant difference test (*p* < 0.05).

**Figure 3:**
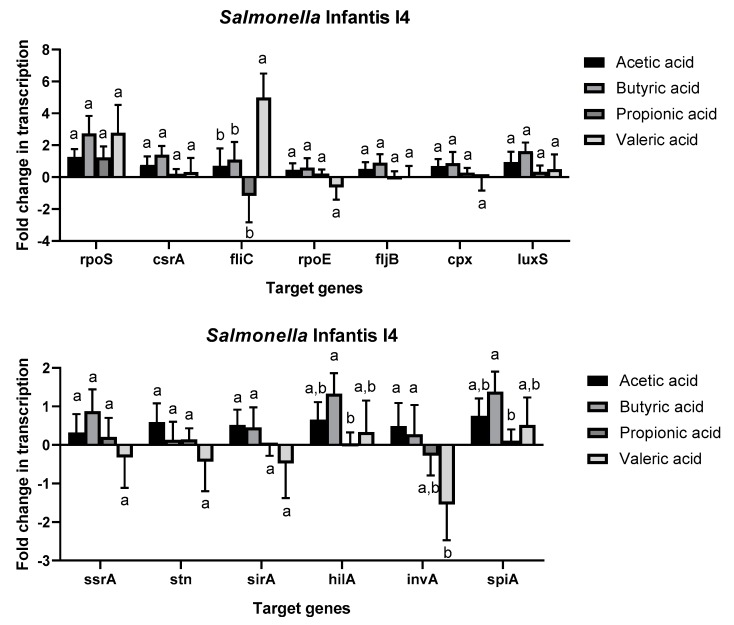
Fold change normalized to control gene *16s rRNA* in the transcription of genes related to virulence and stress in presence of short chain fatty acids in comparison to control TSB growth media in *Salmonella* Infantis I4. Only target genes with different letters between the different growth media present statistically significant differences after analysis of variance (one-way ANOVA) and the Tukey’s honestly significant difference test (*p* < 0.05).

The expression profiles of the two strains were different. *Salmonella* Typhimurium T7 showed overexpression of all genes tested in this study in presence of SCFAs (Figure 2), especially acetic acid and butyric acid, in comparison to control growth media. Butyric acid caused significant upregulation of all genes evaluated in this study in comparison to propionic acid and valeric acid. For gene *invA*, the upregulation in the presence of butyric acid was also significantly higher than in the presence of the other three SCFAs. Only the transcription of *rpoS* was not influenced by SCFAs. *Salmonella* Infantis I4 (Figure 3) showed different expression profiles according to the gene and the SFCAs. It is remarkable that nine genes showed no significant differences in their regulation between the different SCFAs and in comparison to the control sample. While gene *fliC* was significantly upregulated in the presence of valeric acid, gene *invA* was significantly downregulated in comparison to the other SCFAs. The expression of *hilA* and *spiA* was also significantly upregulated in the presence of acetic acid and butyric acid.

Previous studies have shown that acetate can be used by *Salmonella* as a signal for invasion gene expression by upregulation of genes such as *hilA* and *sirA* [17]. On the other side, it has been observed that propionate downregulates invasion genes located in SPI-1 [15]. The results of this study showed that upregulation of *hilA* or *spiA* genes caused by propionate was lower in the presence of propionic acid than in the presence of other SCFAs, such as acetic acid or butyric acid. In this regard, it is worth mentioning that most of the genes tested in this study were significantly upregulated in *S.* Typhimurium T7 in the presence of acetic acid and especially butyric acid (Figure 2). In concordance with these results, it was observed that enterohemorrhagic *E. coli* increased the expression of virulence genes in the presence of subinhibitory concentrations of SFCAs and especially in the presence of butyric acid [14]. Consequently, subinhibitory concentrations of SCFAs can produce an overexpression of virulence genes in common enteric pathogens, such as *Salmonella* and *E. coli*, resulting in increased virulence of these pathogens.

In previous work, organic acids, such as acetic acid, showed anti-quorum sensing activity in *Salmonella* and *E. coli* [24]. Quorum sensing is an important mechanism that regulates the expression of virulence determinants to effectively colonize the host [34]. In this study, the expression of *luxS*, which codifies the Autoinducer-2 (AI-2), was significantly upregulated in *S.* Typhimurium T7 in the presence of SCFAs. The authors consider that SCFAs can inhibit the quorum sensing mechanism by blocking AI-2 instead of downregulating the genes implicated in the production of the signal molecule. Finally, it is necessary to re-emphasize the different transcriptional profiles observed in *S.* Typhimurium T7 and *S.* Infantis I4. This is due to the strain variability in the behavior of foodborne pathogens [35]. It is important to consider that *S.* Typhimurium is one of the most pathogenic serotypes of *S. enterica* [36]. As a consequence, strains of this serotype could have an enhanced virulence response in the presence of subinhibitory concentrations of SCFAs.

## 3. Materials and Methods

### 3.1. Salmonella enterica Strains and Short Chain Fatty Acids

A total of twelve *Salmonella* strains belonging to seven different serotypes and two *Salmonella* subspecies were included in this study (Table 2). All the strains were previously isolated from poultry houses and chicken meat in our laboratory according to ISO 6579:2003 [37]. The strains were kept at −20 °C in tryptic soy broth (TSB, Oxoid, UK) supplemented with 20% glycerol until use. *Salmonella* strains were previously grown in nutrient agar (Applichem Panreac, Barcelona, Spain) and incubated for 24 h at 37 °C before use. The following four different SCFAs were tested in this study: acetic acid (≥99%, Sigma Aldrich, Schnelldorf, Germany), butyric acid (≥99%, Alfa Aesar, ThermoFisher Scientific, Massachusetts, Waltham, MA, USA), propionic acid (≥99%, Sigma Aldrich), and valeric acid (≥99%, Sigma Aldrich).

### 3.2. Minimum Inhibitory Concentration and Minimum Biocidal Concentration of Short Chain Fatty Acids

Minimum inhibitory concentrations (MICs) of SCFAs were determined according to the microdilution broth method described by the Clinical and Laboratory Standards Institute (CLSI) guidelines. An initial concentration of SCFAs at 20% in Mueller–Hinton broth was used to perform serial dilutions of the tested SCFAs. *Salmonella* strains were initially grown in nutrient agar for 24 h at 37 °C, and isolated colonies were used to prepare a saline suspension with a turbidity equivalent to a 0.5 McFarland standard. Two serial dilutions of the initial inoculum in Mueller–Hinton broth were performed to obtain a final concentration of 1 × 10^6^ CFU/mL. In a 96-well microtiter plate, 100 µL of each dilution was mixed with 100 µL of the final inoculum, and microplates were incubated for 24 h at 37 °C. The MIC was defined as the lowest concentration in which no visual bacterial growth was observed. The liquid of wells with no visual bacterial growth observed was transferred to nutrient agar plates to determine the minimum bactericidal concentration (MBC). Plates were incubated for 24 h at 37 °C.

### 3.3. Motility Assays

Motility assays were carried out in nutrient semisolid agar plates composed of 8 g/L of nutrient broth (AppliChem, Panreac, Barcelona, Spain), 4 g/L of agar (Liofilchem, Abruzzi TE, Italy), and 0.05 g/L of 2,3,5-triphenyltetrazolium chloride (Sigma Aldrich). Five different types of agar were used: a control agar and four semisolid agars, each supplemented with one SCFA at the highest subinhibitory concentration observed in this study. A saline suspension with a turbidity equivalent to 0.5 McFarland was prepared for each strain from the isolated colonies of nutrient agar plates incubated for 24 h at 37 °C. An inoculating loop was immersed in the *Salmonella* saline suspension, and then the strains were inoculated in the semisolid agar plates by stabbing. Motility agar plates were incubated at 37 °C for 48 h. After incubation, the ratio between the stabbing point and the end of the growth circle was measured. The experiments were carried out in triplicate.

### 3.4. Biofilm Formation on Polystyrene

The effect of SCFAs on the ability to produce a biofilm in polystyrene of *Salmonella* strains included in this study was evaluated according to the method described by Stepanović et al. [38]. Five different growth media were used: control TSB media and four growth media, each supplemented with one of the SCFAs tested in this study to a final concentration of the highest subinhibitory concentration observed the MICs evaluation. Polystyrene, 96-well microplates were filled with 200 µL of the corresponding growth media and then inoculated with 20 µL of saline solution with a *Salmonella* concentration of 10^4^ CFU/mL. Microplates were incubated for 24 h at 37 °C and then washed three times with 250 µL of distilled water. *Salmonella* cells that were adhered to the surface of the wells were fixed with 250 µL of methanol for 15 min. Then, wells were emptied, air dried, and filled with 250 µL of 0.1% crystal violet solution (Panreac, Barcelona, Spain) for 5 min. Excess crystal violet was removed under tap water, and the crystal violet that was adhered to the wells was resolubilized with 250 µL of 33% acetic acid solution. The absorbance of the microplates was read at 630 nm using a plate reader (das, Palombara Sabina, Italy). The experiments were carried out in triplicate.

### 3.5. RNA Isolation and RT-qPCR

The transcription of 14 *Salmonella* genes (Table 3) in the presence of subinhibitory concentrations of SCFAs was evaluated in two *Salmonella* strains (*S.* Typhimurium T7 and *S.* Infantis I4). A total of five growth media were used: TSB media that was used as control media and four growth media, each supplemented with the highest subinhibitory concentrations of one of the SFCAs tested in this study. Fifteen-milliliter plastic tubes were filled with 10 mL of the corresponding growth media and 100 µL of a saline solution with a concentration of 10^4^ CFU/mL of the corresponding *Salmonella* strains. The tubes were incubated for 12 h at 37 °C and then centrifuged at 2000× *g* for 10 min. The supernatant was discarded, and the pellet was resuspended in 1 mL of NZYol (nzytech, Lisboa, Portugal), and the RNA was isolated according to the manufacturer’s recommendations. Immediately, RNA was quantified using a fluorometer (Qubit, Invitrogen, Carlsbad, CA, USA, Thermofisher Scientific) and reversed transcribed using the NZY First-Strand cDNA Synthesis kit (nzytech) following the manufacturer’s protocol. The cDNA was stored at −20 °C until use.

The 10 µL RT-qPCR reactions, composed of 5 µL of NZYSpeedy qPCR green master mix 2× ROX (nzytech), 0.4 µL of each primer, 1 µL of the sample, and 3.2 µL of RNase-free water, were carried out in a QuantStudio 12 k Flex real-time PCR system (Applied Biosystems, ThermoFisher Scientific, Foster, CA, USA). The conditions were as follows: initial denaturation at 95 °C for 20 s, followed by 40 cycles of denaturation at 95 °C for 1 s, annealing at 60 °C for 20 s, and a final melting curve program of 15 s at 95 °C, 60 s at 60 °C, and followed by a dissociation step for 15 s at 95 °C. The expression of target genes in comparison to the control gene 16 s rRNA was evaluated using the 2^ΔΔCt^ method, where ΔΔC_t_ = (Ct_target genes_ − Ct_16s rRNA_)_treatment_ − (Ct_target genes_ − Ct_16s rRNA_)_control._

### 3.6. Statistical Analysis

GraphPad Prism 8 (GraphPad, CA, USA) was used for the statistical analyses. Analysis of variance (one-way ANOVA) and the Tukey’s honestly significant difference test (*p* < 0.05) were used to determine the influence of SCFAs on *Salmonella* motility, biofilm formation, and transcription profiles.

## 4. Conclusions

The results of this study showed that SCFAs are promising antimicrobial substances that can be potentially used for human health and food safety purposes. A SCFA concentration of 3750 µg/mL is enough to inhibit the growth of *Salmonella* strains. In addition, subinhibitory concentrations of SCFAs caused a reduction in motility and biofilm formation in *Salmonella* strains. This study demonstrated that subinhibitory concentrations of SCFAs can enhance the expression of virulence genes in *Salmonella* strains, resulting in a higher virulence of strains. Thus, for future applications in human health and food safety, it is important to assess the appropriate concentrations of the SCFAs used.

## Figures and Tables

**Table 1:** Influence of the four short chain fatty acids tested in this study (acetic acid, propionic acid, butyric acid, and valeric acid) in the motility of *Salmonella* strains at 37 °C expressed in millimeters. Different letters in the same row represent statistically significant differences (*p* < 0.05) between the different agar media.

		Motility (mm)				
Strain	Code	Control (*n* = 3) Mean ± SD	Acetic Acid (*n* = 3) Mean ± SD	Butyric Acid (*n* = 3) Mean ± SD	Propionic Acid (*n* = 3) Mean ± SD	Valeric Acid (*n* = 3) Mean ± SD
*S. enterica* subsp. *arizonae*	AZ3	16.00 ± 1.73 ^a^	2.33 ± 1.53 ^c^	6.00 ± 1.73 ^b,c^	5.00 ± 1.00 ^b,c^	8.67 ± 1.15 ^b^
*S. enterica* subsp. *arizonae*	AZ26	17.00 ± 2.00 ^a^	2.00 ± 1.00 ^c^	5.33 ± 1.53 ^b,c^	5.67 ± 2.10 ^b,c^	8.00 ± 2.00 ^b^
*S*. Bredeney	BR1	15.33 ± 1.15 ^a^	2.00 ± 1.73 ^c^	4.67 ± 1.15 ^c^	3.00 ± 1.00 ^c^	8.33 ± 1.53 ^b^
*S*. Infantis	I18	15.33 ± 2.31 ^a^	1.67 ± 1.15 ^c^	3.67 ± 1.15 ^c^	3.67 ± 0.57 ^c^	8.67 ± 1.15 ^b^
*S*. Infantis	I4	15.33 ± 1.53 ^a^	3.00 ± 1.73 ^c^	5.67 ± 1.15 ^b,c^	2.67 ± 1.53 ^c^	7.33 ± 2.08 ^b^
*S*. Isangi	IG1	15.67 ± 2.08 ^a^	1.67 ± 0.58 ^c^	4.00 ± 1.00 ^c^	4.67 ± 1.15 ^c^	9.00 ± 1.73 ^b^
*S.* Montevideo	M1	20.00 ± 2.00 ^a^	1.33 ± 0.58 ^c^	6.33 ± 2.08 ^b^	5.33 ± 0.58 ^b^	8.33 ± 0.58 ^b^
*S*. Newport	N6	18.33 ± 1.53 ^a^	2.67 ± 1.53 ^d^	7.00 ± 1.73 ^b,c^	4.00 ± 2.00 ^c,d^	10.00 ± 2.00 ^b^
*S*. Typhimurium	T1	20.00 ± 1.00 ^a^	3.67 ± 1.15 ^c^	4.67 ± 1.53 ^c^	6.00 ± 1.73 ^c^	10.00 ± 1.00 ^b^
*S*. Typhimurium	T3	17.77 ± 2.52 ^a^	2.67 ± 0.58 ^c^	4.33 ± 1.53 ^b,c^	4.00 ± 2.65 ^b,c^	8.00 ± 1.00 ^b^
*S*. Typhimurium	T7	19.33 ± 2.08 ^a^	3.33 ± 1.15 ^c^	5.33 ± 1.53 ^c^	3.33 ± 1.15 ^c^	10.67 ± 1.53 ^b^
*S*. Typhimurium	T23	19.67 ± 2.10 ^a^	2.33 ± 2.31 ^c^	6.00 ± 1.00 ^b,c^	3.67 ± 2.10 ^c^	9.33 ± 2.52 ^b^
Total	-	17.36 ± 2.41 ^a^	2.43 ± 1.36 ^d^	4.12 ± 1.60 ^c^	4.24 ± 1.69 ^c^	8.45 ± 1.92 ^b^

**Table 2:** List of *Salmonella* strains included in this study and its source.

Strain	Code	Source
*S. enterica* subsp. *arizonae*	AZ3	Poultry farm
*S. enterica* subsp. *arizonae*	AZ26	Poultry farm
*S*. Bredeney	BR1	Poultry farm
*S*. Infantis	I18	Poultry farm
*S*. Infantis	I4	Poultry farm
*S*. Isangi	IG1	Poultry farm
*S.* Montevideo	M1	Poultry farm
*S*. Newport	N6	Poultry farm
*S*. Typhimurium	T1	Chicken meat
*S*. Typhimurium	T3	Chicken meat
*S*. Typhimurium	T7	Poultry farm
*S*. Typhimurium	T23	Poultry farm

**Table 3:** Primers sequences of genes tested in this study.

Target Genes	Sequence (5′–3′)	Reference
*16s rRNA*	F: AGGCCTTCGGGTTGTAAAGT R: GTTAGCCGGTGCTTCTTCTG	[39]
*luxS*	F: ATGCCATTATTAGATAGCTT R: GAGATGGTCGCGCATAAAGCCAGC	[40]
*hilA*	F: AATGGTCACAGGCTGAGGTG R: ACATCGTCGCGACTTGTGAA	[41]
*invA*	F: CGCGCTTGATGAGCTTTACC R: CTCGTAATTCGCCGCCATTG	[41]
*rpoS*	F: CAAGGGGAAATCCGTAAACCC R: GCCAATGGTGCCGAGTATC	[42]
*csrA*	F: CTGGACTGCTGGGATTTTTC R: CATGATTGGCGATGAGGTC	[43]
*fliC*	F: CTCGGCTACTGGTCTTGGTG R: CCGTAACGGTAACTTTGGCG	[44]
*ssrA*	F: CGGCTGGTATTCTTGTAAGGGT R: AAGCAGACACAAATTCGCAAG	[45]
*stn*	F: CAACCAGATAGTAAAGACCG R: ATTAGCGTAGAGGCAAAAGA	[46]
*fadA*	F: ATCTCTCCGCCCACTTAATGCGTA R: AGCCTTGCTCCAGCGTTTGTTGTA	[42]
*sirA*	F: CCAGCTACTTTCGCAGCAA R: AACACGTTGTAACGCGGTTG	[41]
*spiA*	F: AGGCGCTTGATATGTGC R: GCAGGCTCCGGAATTTTAGG	[46]
*cpx*	F: CATTTAACGACCGCGAGCTG R: ACCCGGATTAAGGCTTAGCG	[44]
*rpoE*	F: CACCTTACGGGAGCTGGATG R: GAAGATACGTGAACGCACCG	[44]
*flijB*	F: ATGGTACTACACTGGATGTATCG R: GTAAAGCCACCAATAGTAAC	[44]
